# Effectiveness of antipsychotic drugs in schizophrenia: a 10-year retrospective study in a Korean tertiary hospital

**DOI:** 10.1038/s41537-020-00122-3

**Published:** 2020-11-19

**Authors:** Sanghoon Oh, Tae Young Lee, Minah Kim, Se Hyun Kim, Suehyun Lee, Sunwoo Cho, Ju Han Kim, Jun Soo Kwon

**Affiliations:** 1 Department of Psychiatry, Seoul National University College of Medicine, Seoul, Republic of Korea; 2 Department of Neuropsychiatry, Seoul National University Hospital, Seoul, Republic of Korea; 3 Seoul National University Biomedical Informatics (SNUBI), Division of Biomedical Informatics, Seoul National University College of Medicine, Seoul, Republic of Korea; 4 Department of Biomedical Informatics, College of Medicine, Konyang University, Daejeon, Republic of Korea; 5 Management Information System, School of Business, Seoul National University, Seoul, Republic of Korea; 6 Department of Brain and Cognitive Sciences, Seoul National University College of Natural Sciences, Seoul, Republic of Korea

**Keywords:** Schizophrenia, Pharmacology

## Abstract

Extensive research has been carried out on the comparative effectiveness of antipsychotic medications. Most studies, however, have been performed in Western countries. The purpose of this study was to compare the effectiveness, indicated by time to any-cause discontinuation, of antipsychotic drugs in a large number of patients with schizophrenia in South Korea. We identified 1458 patients with schizophrenia or schizophreniform disorder who were treated with antipsychotic medications using a clinical data warehouse at the Seoul National University Hospital between March 2005 and February 2014. Kaplan–Meier survival analyses were used to estimate the time to discontinuation of antipsychotic drugs. We compared the survival curves of different antipsychotics using log-rank tests. Overall, the median time to discontinuation for any cause was 133 days (95% CI, 126–147). The longest time to discontinuation was observed for clozapine, followed by aripiprazole, paliperidone, olanzapine, amisulpride, risperidone, quetiapine, ziprasidone, and haloperidol. Specifically, clozapine was significantly different from all other antipsychotic drugs (all *p* < 0.001). Aripiprazole also had a significantly longer time to discontinuation than amisulpride (*p* = 0.001), risperidone (*p* < 0.001), quetiapine (*p* < 0.001), ziprasidone (*p* < 0.001), and haloperidol (*p* < 0.001). In Asian patients with schizophrenia, clozapine was the most effective antipsychotic in terms of time to discontinuation, followed by aripiprazole. This study extends the findings of previous effectiveness studies from Western populations and suggests the need to develop guidelines for the pharmacotherapy of schizophrenia tailored to Asian individuals.

## Introduction

Pharmacotherapy with antipsychotic medication is a keystone of treatment for schizophrenia. Since the introduction of chlorpromazine in the 1950s, many antipsychotics have been developed, and 15–40 of these drugs are available across the world^1,2^. Along with this growth in antipsychotics, the comparative effectiveness of antipsychotic treatment has become a major area of interest within the field of clinical psychiatry. Several meta-analyses have shown that some antipsychotics, such as clozapine and amisulpride, are more efficacious than others^3,4^. However, antipsychotic efficacy measured by symptom scales is not sufficient to reflect the effectiveness of antipsychotics for the treatment of schizophrenia in real-world clinical practice^5,6^.

Time to all-cause medication discontinuation is widely used in the psychiatric field as a proxy measure of antipsychotic effectiveness because it integrates both patient and clinician perspectives on drug efficacy, safety, and tolerability^7^. There have been numerous reports regarding the time to medication discontinuation among antipsychotic drugs^8–12^. The Clinical Antipsychotics Trials of Intervention Effectiveness (CATIE) project, a large, randomized, double-blind trial, is the most famous of comparative studies on the effectiveness of antipsychotics. CATIE investigators found that olanzapine had a significantly longer time to all-cause discontinuation than quetiapine or risperidone but not perphenazine or ziprasidone^13^. Since the CATIE study, the time to discontinuation of antipsychotic drugs has been mainly investigated in naturalistic treatment settings. A recent nationwide cohort study of 29,823 patients with schizophrenia in Sweden reported that clozapine and long-acting injectable (LAI) antipsychotics were the most effective in preventing treatment discontinuation^14^.

However, there has been relatively little information in Asian populations regarding the comparative effectiveness of antipsychotic medications. The issue of ethno-psychopharmacology, defined as dramatic cross-ethnic variations in dosing and side effects in response to psychotropics, has received considerable critical attention^15^. Previous studies have investigated the influence of ethnicity on the metabolism^16^, pharmacogenetics^17^, dosing^18^, and side effects of antipsychotic medications^19^ in addition to medication adherence^20^. Ethnic differences in these factors may eventually lead to differences in antipsychotic effectiveness or overall treatment response^9,21,22^. For example, Azekawa et al.^9^ suggest that aripiprazole is more effective than other antipsychotics, such as olanzapine and risperidone, in Japanese patients with schizophrenia or schizoaffective disorder. This finding was somewhat contradictory to the existing Western literature.

The aim of this study was to try and establish the comparative effectiveness of antipsychotic medications for the prevention of treatment discontinuation in our Asian population. We hypothesized that the hierarchy for the effectiveness of antipsychotic drugs in our sample would be different from in Western countries. We conducted this 10-year retrospective study to investigate the effectiveness of widely used antipsychotic medications (one typical and eight atypical antipsychotics) in patients with schizophrenia using a large, naturalistic clinical sample in South Korea, with time to all-cause discontinuation as the comparator outcome.

## Results

### Sample description

This study included 3257 antipsychotic episodes of 1458 patients who were prescribed oral antipsychotic medications between 1 March 2005, and 28 February 2014. Patients had an average of 2.2 antipsychotic episodes during the study period. Of 1458 patients, 32.5% had one antipsychotic episode, 34.9% had two antipsychotic episodes, 17.5% had three antipsychotic episodes, and 15.1% had four or more antipsychotic episodes. The patient characteristics of each antipsychotic group are shown in Table 1. The most frequently prescribed antipsychotic medication was risperidone (*n* = 730), followed by aripiprazole (*n* = 556) and olanzapine (*n* = 441) during the study period. Haloperidol (*n* = 83) was the least frequently used drug. In terms of the time trend of oral antipsychotic prescription, the use of risperidone decreased dramatically over time. This position was replaced by paliperidone with a relatively high proportion in the most recent years. Aripiprazole consistently occupied a considerable proportion regardless of the year, and olanzapine also remained third on average (Supplementary Fig. 1 in the Supplementary Material).

**Table 1:** Demographic and clinical characteristics of the study sample by antipsychotic medication.

	Amisulpride	Aripiprazole	Clozapine	Haloperidol	Olanzapine	Paliperidone	Quetiapine	Risperidone	Ziprasidone
	(*N* = 213)	(*N* = 556)	(*N* = 336)	(*N* = 83)	(*N* = 441)	(*N* = 393)	(*N* = 355)	(*N* = 730)	(*N* = 150)
Age at baseline, years, *n* (%)									
≤18	11 (5.2)	95 (17.1)	27 (8.0)	3 (3.6)	36 (8.2)	22 (5.6)	15 (4.2)	58 (8.0)	11 (7.3)
19–34	133 (62.4)	309 (55.6)	199 (59.2)	43 (51.8)	243 (55.1)	250 (63.6)	205 (57.8)	419 (57.4)	109 (72.7)
35–49	49 (23.0)	124 (22.3)	95 (28.3)	32 (38.6)	114 (25.9)	88 (22.4)	89 (25.1)	191 (26.2)	25 (16.7)
50–64	16 (7.5)	24 (4.3)	13 (3.9)	4 (4.8)	42 (9.5)	26 (6.6)	32 (9.0)	47 (6.4)	3 (2.0)
≥65	4 (1.9)	4 (0.7)	2 (0.6)	1 (1.2)	6 (1.4)	7 (1.8)	14 (3.9)	15 (2.1)	2 (1.3)
mean (SD)	32.2 (11.8)	28.8 (10.9)	30.8 (10.2)	33.3 (10.4)	32.3 (11.8)	31.3 (11.1)	33.8 (12.9)	32.3 (11.8)	28.7 (9.3)
Male, *n* (%)	122 (57.3)	245 (44.1)	189 (56.3)	47 (56.6)	217 (49.2)	195 (49.6)	163 (45.9)	376 (51.5)	65 (43.3)
Diagnosis, *n* (%)									
Schizophrenia	212 (99.5)	542 (97.5)	335 (99.7)	83 (100)	429 (97.3)	380 (96.7)	349 (98.3)	721 (98.8)	148 (98.7)
Schizophreniform	1 (0.5)	14 (2.5)	1 (0.3)	0 (0)	12 (2.7)	13 (3.3)	6 (1.7)	9 (1.2)	2 (1.3)
Comorbid depression^a^, *n* (%)	6 (2.8)	14 (2.5)	5 (1.5)	0 (0)	15 (3.4)	10 (2.5)	9 (2.5)	12 (1.6)	4 (2.7)

^a^Depression includes major depressive disorder, single episode, severe without psychotic features (International Classification of Diseases, 10th edition [ICD-10] code of 32.2) and single episode, unspecified (ICD-10 code of 32.9).

The mean daily doses in each antipsychotic group were as follows: 356.8 ± 298.0 mg for amisulpride; 14.0 ± 9.4 mg for aripiprazole; 252.7 ± 136.2 mg for clozapine; 7.5 ± 7.8 mg for haloperidol; 13.2 ± 9.1 mg for olanzapine; 7.2 ± 5.6 mg for paliperidone; 224.4 ± 254.4 mg for quetiapine; 3.0 ± 2.1 mg for risperidone; and 72.8 ± 41.0 mg for ziprasidone. The average daily antipsychotic doses in Korean patients are generally within or slightly below the target dose range recommended by the international consensus study^23^.

### Discontinuation of treatment

**Fig. 1:**
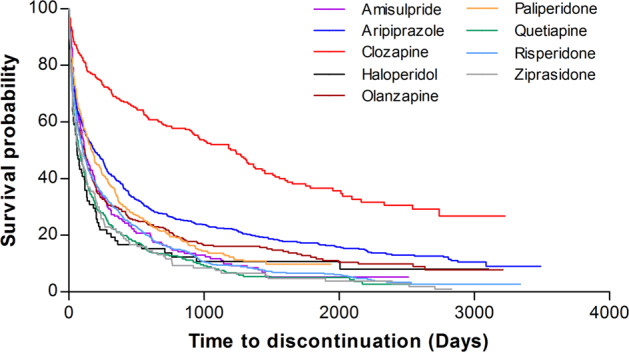
Kaplan–Meier time to discontinuation curves by antipsychotic medication. Median overall time to discontinuation for any cause was 133 (95% CI, 126–147) days. The ranking of the longest time to discontinuation was clozapine, aripiprazole, paliperidone, olanzapine, amisulpride, risperidone, quetiapine, ziprasidone, and haloperidol.

Of a total of 3257 antipsychotic episodes, 2659 (81.6%) were discontinued during the study period, with the median time to discontinuation for each antipsychotic drug shown in Table 2. Kaplan–Meier survival curves for time to discontinuation by antipsychotic medication are displayed in Fig. 1. It is apparent from this figure that at any point along the follow-up time, the survival probability of clozapine is consistently higher than that of all other antipsychotics. The log-rank test revealed significant differences in time to all-cause discontinuation among antipsychotic drugs (Table 3). Clozapine was found to have a significantly longer time to discontinuation than all other antipsychotics (all *p* < 0.001). Even after a Bonferroni correction was applied, clozapine and aripiprazole differed significantly from other antipsychotic medications in the time to discontinuation. For example, aripiprazole had a significantly longer time to discontinuation than amisulpride (*χ*^2^ = 11.8, df = 1, *p* = 0.001), risperidone (*χ*^2^ = 31.3, df = 1, *p* < 0.001), quetiapine (*χ*^2^ = 42.2, df = 1, *p* < 0.001), ziprasidone (*χ*^2^ = 26.7, df = 1, *p* < 0.001), and haloperidol (*χ*^2^ = 12.2, df = 1, *p* < 0.001). Although the median time to discontinuation of haloperidol was the shortest, no statistically significant difference was observed among amisulpride, risperidone, quetiapine, ziprasidone, and haloperidol. Furthermore, the above results remained similar in an exploratory analysis in which we excluded the 1514 overlapping antipsychotic episodes to rule out polypharmacy bias (Supplementary Table 1 and Supplementary Fig. 2 in the Supplementary Material).

**Table 2:** Incidence rate of discontinuation and median time to discontinuation by antipsychotic medication.

					Time to discontinuation	
Antipsychotics	Number of patients	Number of discontinuations	Person years	Incidence rate	Median (Days)	95% CI
Amisulpride	213	189	181	1.04	119	(98–175)
Aripiprazole	556	422	716	0.59	175	(133–231)
Clozapine	336	183	804	0.23	1233	(955–1380)
Haloperidol	83	73	67	1.09	61	(38–119)
Olanzapine	441	356	430	0.83	119	(91–133)
Paliperidone	393	318	350	0.91	168	(133–222)
Quetiapine	355	322	245	1.31	78	(60–105)
Risperidone	730	654	640	1.02	104	(89–126)
Ziprasidone	150	142	111	1.28	70	(53–128)

**Table 3:** Pairwise comparison of antipsychotic medications on time to all-cause discontinuation using log-rank tests.

	Significance for antipsychotics:							
Antipsychotics	Amisulpride	Aripiprazole	Clozapine	Haloperidol	Olanzapine	Paliperidone	Quetiapine	Risperidone
Amisulpride								
Aripiprazole	0.001*							
Clozapine	<0.001*	<0.001*						
Haloperidol	0.179	<0.001*	<0.001*					
Olanzapine	0.450	0.002	<0.001*	0.078				
Paliperidone	0.104	0.021	<0.001*	0.009	0.359			
Quetiapine	0.030	<0.001*	<0.001*	0.910	0.001*	<0.001*		
Risperidone	0.622	<0.001*	<0.001*	0.343	0.070	0.010	0.027	
Ziprasidone	0.060	<0.001*	<0.001*	0.845	0.007	<0.001*	0.882	0.078

*Significant after Bonferroni correction for multiple testing.

The incidence rates of all-cause discontinuation are also presented in Table 2. Clozapine had the lowest incidence rate of discontinuation (0.23 per person-years [PYs]), followed by aripiprazole (0.59 per PYs). In contrast, quetiapine, ziprasidone and haloperidol (1.31, 1.28 and 1.09 per PYs, respectively) had relatively high incidence rates of discontinuation.

### Concomitant medications

There were significant differences among the antipsychotic groups in the proportions or types of concomitant medications (Table 4). Clozapine was the most likely to have concomitant mood stabilizers and antidepressants (range 0.3–19.9% and 0.3–30.7%, respectively). Antidepressant use in patients on quetiapine was the lowest (range 0.6–11.3%). The proportions of anxiolytic/hypnotic use had few substantial differences among antipsychotic groups. In the case of anticholinergics/propranolol, haloperidol showed the highest (range 27.7–53.0%), and quetiapine showed the lowest (range 17.7–29.9%) proportion of usage.

**Table 4:** Proportion of concomitant medication use by antipsychotic drug.

	Amisulpride	Aripiprazole	Clozapine	Haloperidol	Olanzapine	Paliperidone	Quetiapine	Risperidone	Ziprasidone
Concomitant medication	(*N* = 213)	(*N* = 556)	(*N* = 336)	(*N* = 83)	(*N* = 441)	(*N* = 393)	(*N* = 355)	(*N* = 730)	(*N* = 150)
Mood stabilizers, *n* (%)									
Carbamazepine	1 (0.5)	2 (0.4)	1 (0.3)	0 (0)	1 (0.2)	2 (0.5)	6 (1.7)	6 (0.8)	2 (1.3)
Lamotrigine	15 (7.0)	29 (5.2)	27 (8.0)	5 (6.0)	17 (3.9)	13 (3.3)	19 (5.4)	22 (3.0)	6 (4.0)
Lithium	9 (4.2)	10 (1.8)	10 (3.0)	6 (7.2)	14 (3.2)	10 (2.5)	15 (4.2)	16 (2.2)	3 (2.0)
Topiramate	2 (0.9)	19 (3.4)	31 (9.2)	0 (0)	16 (3.6)	12 (3.1)	16 (4.5)	16 (2.2)	7 (4.7)
Valproate	25 (11.7)	58 (10.4)	67 (19.9)	11 (13.3)	43 (9.8)	41 (10.4)	45 (12.7)	84 (11.5)	11 (7.3)
Antidepressants, *n* (%)									
Bupropion	8 (3.8)	11 (2.0)	4 (1.2)	0 (0)	8 (1.8)	11 (2.8)	8 (2.3)	14 (1.9)	4 (2.7)
Clomipramine	6 (2.8)	3 (0.5)	10 (3.0)	4 (4.8)	4 (0.9)	1 (0.3)	4 (1.1)	7 (1.0)	0 (0)
Duloxetine	4 (1.9)	5 (0.9)	2 (0.6)	0 (0)	5 (1.1)	7 (1.8)	2 (0.6)	3 (0.4)	1 (0.7)
Escitalopram	43 (20.2)	86 (15.5)	56 (16.7)	7 (8.4)	59 (13.4)	55 (14.0)	40 (11.3)	87 (11.9)	22 (14.7)
Fluoxetine	17 (8.0)	41 (7.4)	35 (10.4)	6 (7.2)	37 (8.4)	25 (6.4)	23 (6.5)	48 (6.6)	15 (10.0)
Fluvoxamine	5 (2.3)	2 (0.4)	6 (1.8)	0 (0)	3 (0.7)	3 (0.8)	2 (0.6)	13 (1.8)	2 (1.3)
Mirtazapine	5 (2.3)	6 (1.1)	1 (0.3)	0 (0)	5 (1.1)	4 (1.0)	8 (2.3)	6 (0.8)	2 (1.3)
Paroxetine	6 (2.8)	10 (1.8)	5 (1.5)	0 (0)	7 (1.6)	3 (0.8)	7 (2.0)	17 (2.3)	1 (0.7)
Sertraline	35 (16.4)	65 (11.7)	103 (30.7)	14 (16.9)	42 (9.5)	61 (15.5)	34 (9.6)	77 (10.5)	22 (14.7)
Venlafaxine	3 (1.4)	2 (0.4)	1 (0.3)	0 (0)	0 (0)	3 0.8)	3 (0.8)	4 (0.5)	0 (0)
Anxiolytics/Hypnotics, *n* (%)									
Alprazolam	21 (9.9)	35 (6.3)	26 (7.7)	4 (4.8)	21 (4.8)	30 (7.6)	23 (6.5)	48 (6.6)	10 (6.7)
Clonazepam	58 (27.2)	122 (21.9)	72 (21.4)	11 (13.3)	100 (22.7)	82 (20.9)	95 (26.8)	142 (19.5)	29 (19.3)
Diazepam	15 (7.0)	25 (4.5)	19 (5.7)	13 (15.7)	18 (4.1)	18 (4.6)	21 (5.9)	45 (6.2)	5 (3.3)
Lorazepam	114 (53.5)	326 (58.6)	211 (62.8)	42 (50.6)	267 (60.5)	213 (54.2)	205 (57.7)	425 (58.2)	86 (57.3)
Trazodone	7 (3.3)	6 (1.1)	0 (0)	2 (2.4)	8 (1.8)	12 (3.1)	13 (3.7)	12 (1.6)	2 (1.3)
Zolpidem	29 (13.6)	37 (6.7)	23 (6.8)	4 (4.8)	36 (8.2)	43 (10.9)	49 (13.8)	49 (6.7)	14 (9.3)
Anticholinergics/Propranolol, *n* (%)									
Benztropine	70 (32.9)	136 (24.5)	96 (28.6)	44 (53.0)	106 (24.0)	128 (32.6)	85 (23.9)	271 (37.1)	38 (25.3)
Trihexyphenidyl	38 (17.8)	102 (18.3)	62 (18.5)	23 (27.7)	87 (19.7)	81 (20.6)	63 (17.7)	154 (21.1)	28 (18.7)
Propranolol	70 (32.9)	195 (35.1)	139 (41.4)	25 (30.1)	137 (31.1)	133 (33.8)	106 (29.9)	193 (26.4)	47 (31.3)

## Discussion

To the best of our knowledge, this is the largest study examining the comparative effectiveness of antipsychotic medications as measured by time to any-cause discontinuation in an Asian sample. The present results indicate that clozapine demonstrated the best effectiveness for treatment continuation among antipsychotics. Apart from clozapine, the following two drugs—aripiprazole and paliperidone—have been shown to be most effective, whereas quetiapine, ziprasidone and haloperidol displayed a relatively short time to discontinuation.

Consistent with previous comparative studies of antipsychotic effectiveness in routine clinical settings^8,11,14^, we also found that clozapine has the longest time to medication discontinuation. Close inspection of these results, however, shows that clozapine is overwhelmingly superior to other antipsychotics in our sample than studies in Western populations. For example, one study by Ascher-Svanum reported that the median time to discontinuation for clozapine and olanzapine was 302.8 and 266 days, respectively^8^, which differed from our results (1233 and 119 days, respectively). These differences could be attributed to our longer follow-up period than in previous studies. It has been well documented that long-term maintenance therapy with clozapine is successful^24^; hence, the longer follow-up may have led to greater differences in time to discontinuation for clozapine and other antipsychotics. Another possible explanation may lie in the interethnic differences in the pharmacodynamics of clozapine. Several studies have suggested that Asian patients can be effectively and safely treated with lower doses of clozapine than Caucasian patients^18,25^. Our results indicating clozapine’s superiority over time to discontinuation extend the findings of previous studies from Western countries and highlight the usefulness of clozapine in long-term maintenance therapy, especially in the Asian population.

Findings from survival analysis illustrate the ranking of the antipsychotic medications in the following descending order: clozapine, aripiprazole, paliperidone, olanzapine, amisulpride, risperidone, quetiapine, ziprasidone, and haloperidol. In line with many previous studies^8,10^, we also observed that second-generation antipsychotics (SGAs) were generally more effective in terms of treatment continuation than first-generation antipsychotics (FGAs). Following correction for multiple comparisons, some SGAs (aripiprazole and clozapine) maintained their initial significant differences from haloperidol, which is FGAs. Interestingly, paliperidone was more effective than risperidone in antipsychotic treatment. Since paliperidone is the primary active metabolite of risperidone^26^, it is believed that paliperidone and risperidone have similar efficacy. However, the comparative effectiveness of paliperidone and risperidone is not well known because few studies have been designed to directly compare the two medications^27,28^. Therefore, our finding that the real-world effectiveness of paliperidone is higher than that of risperidone is meaningful. It seems possible that this result is due to the nature of paliperidone with extended-release formulation, which provides more stable plasma concentrations^29^. However, this result should be interpreted with caution because of the statistically significant difference (*p* = 0.01) between paliperidone and risperidone is lost after Bonferroni correction (required *p*-value < 0.0014).

With respect to the incidence of discontinuation, clozapine was the most effective antipsychotic drug, followed by aripiprazole. As with the survival analysis for time to discontinuation, clozapine and aripiprazole were the top ranked. Taken together, our results indicate that aripiprazole is particularly effective among SGAs. This finding is consistent with a previous study of Japanese patients^9^, in which aripiprazole had a significant or trending longer time to discontinuation than the other five SGAs (olanzapine, risperidone, blonanserin, quetiapine, or perospirone). Surprisingly, these results have not been observed in European and Western/American populations. A possible reason for the superior effectiveness of aripiprazole, especially in Asian populations, may be related to ethnic differences in pharmacological properties. Although there is no significant difference with regard to ethnicity in the treatment response to aripiprazole^30^, some evidence has suggested more favorable safety profiles in Asian patients than in Caucasian patients^19,31^. Since effectiveness takes into account the parameters of efficacy and safety/tolerability, an inter-ethnic difference in the vulnerability for the development of side effects may lead to differences in the effectiveness of aripiprazole. However, these comparisons of the effectiveness of antipsychotics reported in studies in different countries must be interpreted with caution because of differences in the study methodologies, medication variables (e.g., usual dosage and local prescription traditions) and other confounding factors^32^. For example, antipsychotic doses for patients with schizophrenia, the proportion of antipsychotic polypharmacy, and the use of concomitant medications differed across countries/territories^33,34^. Furthermore, a recent large-scale observational study reported that clozapine plus aripiprazole resulted in a better outcome than any other antipsychotic treatment^35^. Considering the findings of this previous study, the higher rank of the time to discontinuation of aripiprazole in our study may also be a consequence of its use in combination with clozapine. Our additional analysis of the comparative effectiveness for antipsychotic monotherapy, however, showed that aripiprazole was still the top-ranked antipsychotic monotherapy among other antipsychotics without the combined use of clozapine. These results seem to suggest that aripiprazole could be recommended as the first-line choice of antipsychotic medication in Asian populations.

Our findings regarding the comparative effectiveness (as measured by all-cause time to discontinuation) of antipsychotic drugs differed somewhat from previous studies conducted in Western countries^14,36,37^. Although we have already discussed that these differences may be explained by ethnicity, other factors contributing to these differences should also be considered. For example, the CATIE trial reported that olanzapine was more effective than other antipsychotic medications in patients with chronic schizophrenia^13^. Furthermore, one study by Bitter et al.^36^ compared the efficacy of olanzapine with clozapine in patients with treatment-resistant schizophrenia and found that olanzapine displayed similar efficacy to clozapine and represented a safe alternative in the management of patients with refractory schizophrenia. In contrast to these findings, a naturalistic study^37^ examining the effectiveness of antipsychotics used to treat first-episode psychosis showed that the ranking of the longest time to discontinuation was risperidone, quetiapine, aripiprazole, and olanzapine. Our analysis including all patients with schizophrenia, regardless of the illness stages or treatment resistance, revealed that olanzapine exhibited a moderate level of effectiveness among the medications studied. The reason for this difference between our results and some published studies may be related to the stages of illness and responsiveness to antipsychotic medication^38^. A further study with more focus on these possible factors is therefore suggested.

Our results showing the proportion of concomitant medication use are generally consistent with a previous pan-Asian study reporting frequent prescriptions of antidepressants (11.7%), mood stabilizers (13.7%), benzodiazepines (27.8%) and anticholinergics (45.6%) along with antipsychotics^33^. The results from our study also showed differences in the use of concomitant medications among antipsychotic groups. In accordance with previous studies^39^, clozapine was the most frequently used with mood stabilizers and antidepressants. Because clozapine is considered to be the gold standard for treatment-resistant schizophrenia^40^, patients taking clozapine usually have a high disease severity and comorbid illness^41^. Therefore, the use of concomitant medications such as mood stabilizers to treat comorbidities is higher in patients on clozapine than in other antipsychotics. On the other hand, it is remarkable that anticholinergics/propranolol was the most frequently used with haloperidol among antipsychotic drugs. This is consistent with previous studies, which reported that FGA-treated patients had more extrapyramidal side effects and used more adjuvant anticholinergics^42,43^.

Several limitations to this study need to be acknowledged. First, we were unable to determine the extent to which individuals defined as having discontinued antipsychotic treatment from our data warehouse actually continued pharmacotherapy at other centers because of relocation or other reasons. This is the inherent limitation of research using electronic databases^10^. Our database also did not include information about potential confounders (e.g., treatment history and duration of illness) associated with medication adherence^44^. Second, we did not limit the antipsychotic episodes to monotherapy; thus, we could not distinguish between the augmenting and augmented antipsychotic medication. Despite the potential for underestimation of time to discontinuation for antipsychotics frequently used as augmenting drugs, it is important to determine the true comparative effectiveness that represents complex treatment regimens in real-world practice. Fortunately, the current main findings were generally consistent with those of further analysis of antipsychotic episodes for monotherapy only. Third, LAIs were not included in the current study. Because LAIs, by their nature, appear to lead to an improvement in treatment continuation^45^, a comparison of the effectiveness between oral antipsychotics and LAIs on the same line may not be adequate. For example, all LAIs except clozapine showed a lower risk of treatment failure than oral antipsychotic medications^14^. Therefore, this study is meaningful in that it excluded the confounding effects that can be caused by differences in drug formulations. Last, our data were derived from a single urban tertiary care hospital and therefore may have limited generalizability to other facilities or countries.

In conclusion, this study extends our knowledge of the comparative effectiveness of antipsychotic treatments in the Asian population. Based on time to discontinuation, clozapine was the most effective antipsychotic medication, as has been shown in Caucasian subjects. In addition, our results suggest that aripiprazole had a significantly longer time to discontinuation than other antipsychotics except clozapine, which indicates that aripiprazole may be the most recommended first-line antipsychotic in Asian patients with schizophrenia. Such information could be used as an aid to develop Asian guidelines for the pharmacotherapy of schizophrenia. However, a further research involving many different Asian countries needs to be undertaken before providing guidelines suitable for Asians.

## Methods

### Study design, sample, and data sources

This naturalistic retrospective study at the Seoul National University Hospital (SNUH) compared the effectiveness of oral antipsychotic medications in patients with schizophrenia. SNUH is a large urban tertiary hospital in Seoul, South Korea. Data were obtained from a Clinical Data Warehouse (CDW) totally synchronized with the electronic medical records (EMR) system generated as part of the usual clinical practice. The SNUH CDW encompasses all routine clinical information, such as the demographics, diagnosis, medication profiles, and laboratory results, from each visit since 2001. Approval from the Institutional Review Board at SNUH was obtained prior to collecting and analyzing the data. Written informed consent is not required for CDW-based studies using anonymized data.

The study population included all patients who were treated in the inpatient and/or outpatient setting at SNUH from 1 March 2005 to 28 February 2014, with a diagnosis of schizophrenia or schizophreniform disorder (*International Statistical Classification of Diseases and Related Health Problems, Tenth Revision [ICD-10]* codes: 20, 200, 201, 203, 205, and 208). For the homogeneity of the study population, patients with schizoaffective disorder or bipolar disorder (*ICD-10* codes: 25, 25X, 30, 30X, 31, and 31X) were excluded. From this sample, we selected patients prescribed any one of the following antipsychotic medications: amisulpride, aripiprazole, clozapine, haloperidol, olanzapine, paliperidone, quetiapine, risperidone, and ziprasidone. Here, we included only the oral form of the above drugs and not the LAI formulation.

Using the treatment episodes approach, we defined an antipsychotic episode as the sustained prescription period of any antipsychotic medications during the study. A patient could have several antipsychotic episodes, with the possibility of overlap between these antipsychotic episodes throughout the 10-year study. This treatment episode approach provides overall information on a variety of treatment courses per patient from the perspective of the longitudinal treatment of the illness^8^.

All patient prescription data derived from the SNUH CDW were handled by bioinformatics experts using Python programming. Medication information was extracted according to the generic name of drugs instead of the brand names or different prescription codes because all medications have different drug codes depending on the dose of the drug.

### Outcomes

The primary outcome measure in this study was the discontinuation of antipsychotic medication. All-cause discontinuation, namely, treatment discontinuation for any cause, including a lack of efficacy, intolerable side effects, and the clinician’s decision to stop the medication^46^, was used in our analysis. We defined discontinuation as the period longer than 56 days during which the patients did not have any medication supply. Time to discontinuation was calculated as the duration (in days) of the antipsychotic medication episode, a continuous period of medication supply from the day of its first prescription until the date of discontinuation or the end of the study period.

Other outcomes were the incidence rate of antipsychotic discontinuation and proportion of concomitant medication use. The incidence rate measured the occurrence of discontinuation in each antipsychotic group per unit of time (person-years) of follow-up. On the other hand, we measured concomitant medications used more than once during a continuous period of each antipsychotic possession (i.e., time to discontinuation of antipsychotic medication). Concomitant medications belonging to the following four categories were used for the analysis: mood stabilizers, antidepressants, anxiolytics/hypnotics, and anticholinergics/propranolol.

### Statistical analyses

We first calculated descriptive statistics about sample characteristics, antipsychotic prescription patterns over time, and the proportion of concomitant medications. Second, we estimated the median time to discontinuation for each antipsychotic medication using Kaplan–Meier survival curves. To determine the effectiveness of antipsychotic treatments, we compared the survival curves of each antipsychotic drug by applying log-rank tests for significance. The results from the log-rank tests were corrected for multiple comparisons using Bonferroni correction. The Bonferroni-corrected *p*-value corresponding to a statistical significance level of 0.05 with 36 pairwise comparisons was 0.05/36 = 0.0014. Additional analyses were conducted to see whether the results would be consistent under the condition after removing the polypharmacy effect. For this, we eliminated all antipsychotic episodes where the prescribed antipsychotics overlapped and then performed a survival analysis with only the remaining antipsychotic episodes corresponding to monotherapy. Third, the incidence rate was calculated using the number of patients with antipsychotic discontinuation divided by the total time of follow-up of each patient. Statistical analyses were performed with R (×64, 3.53 version).

### Reporting summary

Further information on research design is available in the Nature Research Reporting Summary linked to this article.

## Supplementary information

Supplementary Material

Reporting Summary

## Data Availability

The data that support the results of this study are available from the corresponding author upon reasonable request. The data are not publicly available because they contain information that might compromise the privacy of the research participants.

## Code availability

The MATLAB and R scripts for data processing and statistical analysis respectively in this study are available from the corresponding author upon reasonable request.
