# Supplementary material for: Effectiveness of antipsychotic drugs in schizophrenia: a 10-year retrospective study in a Korean tertiary hospital
Source: NPJ Schizophr. 2020 Nov 19;6:32. doi: 10.1038/s41537-020-00122-3 (PMC7677553; doi:10.1038/s41537-020-00122-3)
Supplement: Supplementary file 1 — Supplementary Material [file 41537_2020_122_MOESM1_ESM.pdf]

Supplementary Material

Supplementary Figure 1. Time trend of 9 antipsychotic drug uses among patients with schizophrenia.

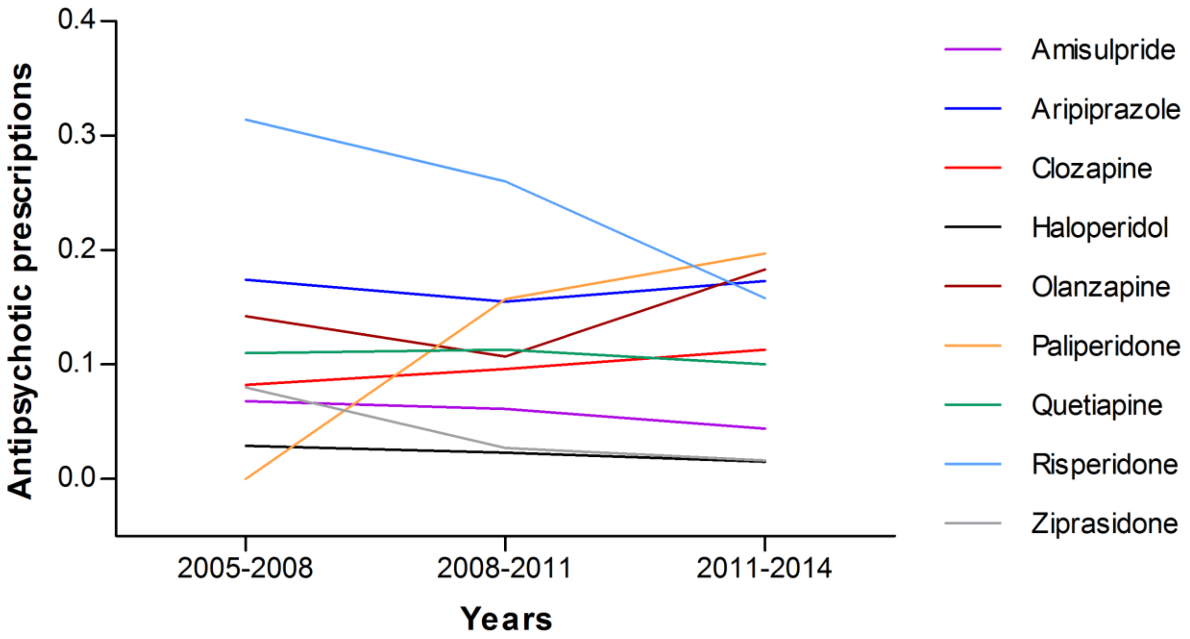

Supplementary Figure 2. Kaplan-Meier time to discontinuation curves by antipsychotic medication administered as monotherapy.

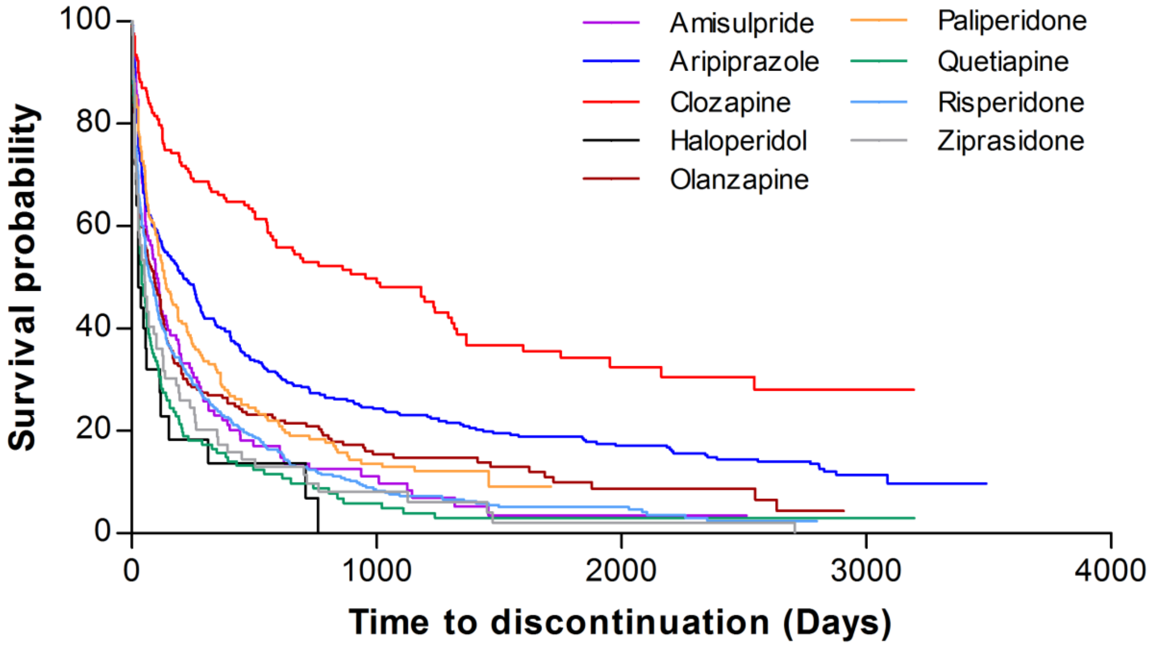

Supplementary Table 1. Incidence rate of discontinuation and median time to discontinuation by antipsychotic medication administered as monotherapy.

Antipsychotics	Number of patients	Number of discontinuations	Person-years	Incidence rate	Time to discontinuation	
					Median (Days)	95% CI
Amisulpride	111	100	80	1.25	103	(63-157)
Aripiprazole	370	312	649	0.48	210	(147-276)
Clozapine	169	97	382	0.25	955	(590-1318)
Haloperidol	25	23	9	2.56	28	(21-118)
Olanzapine	229	189	198	0.96	91	(58-120)
Paliperidone	215	171	171	1.00	133	(108-189)
Quetiapine	137	127	72	1.764	42	(32-63)
Risperidone	416	378	299	1.264	73	(57-102)
Ziprasidone	71	68	44	1.545	53	(28-102)
