# Supplementary material for: Effectiveness of antipsychotic drugs in schizophrenia: a 10-year retrospective study in a Korean tertiary hospital
Source: NPJ Schizophr. 2020 Nov 19;6:32. doi: 10.1038/s41537-020-00122-3 (PMC7677553; doi:10.1038/s41537-020-00122-3)
Supplement: Supplementary file 2 — Reporting Summary [file 41537_2020_122_MOESM2_ESM.pdf]

nature research

Corresponding author(s): Jun Soo Kwon

Last updated by author(s): Aug 14, 2020

Reporting Summary

Nature Research wishes to improve the reproducibility of the work that we publish. This form provides structure for consistency and transparency in reporting. For further information on Nature Research policies, see our Editorial Policies and the Editorial Policy Checklist.

Statistics

For all statistical analyses, confirm that the following items are present in the figure legend, table legend, main text, or Methods section.

n/a	Confirmed	
☐	☒	The exact sample size (*n*) for each experimental group/condition, given as a discrete number and unit of measurement
☐	☒	A statement on whether measurements were taken from distinct samples or whether the same sample was measured repeatedly
☐	☒	The statistical test(s) used AND whether they are one- or two-sided<br>*Only common tests should be described solely by name; describe more complex techniques in the Methods section.*
☐	☒	A description of all covariates tested
☐	☒	A description of any assumptions or corrections, such as tests of normality and adjustment for multiple comparisons
☐	☒	A full description of the statistical parameters including central tendency (e.g. means) or other basic estimates (e.g. regression coefficient) AND variation (e.g. standard deviation) or associated estimates of uncertainty (e.g. confidence intervals)
☐	☒	For null hypothesis testing, the test statistic (e.g. *F*, *t*, *r*) with confidence intervals, effect sizes, degrees of freedom and *P* value noted<br>*Give P values as exact values whenever suitable.*
☒	☐	For Bayesian analysis, information on the choice of priors and Markov chain Monte Carlo settings
☐	☒	For hierarchical and complex designs, identification of the appropriate level for tests and full reporting of outcomes
☒	☐	Estimates of effect sizes (e.g. Cohen's *d*, Pearson's *r*), indicating how they were calculated

Our web collection on statistics for biologists contains articles on many of the points above.

Software and code

Policy information about availability of computer code

Data collection	Data were extracted from a Clinical Data Warehouse (CDW) at the Seoul National University Hospital (SNUH) by the use of Excel software. This database in not open, but anonymous data can be provided to other researchers by contacting the corresponding author.
Data analysis	Statistical analyses were performed with R (x64, 3.53 version).

For manuscripts utilizing custom algorithms or software that are central to the research but not yet described in published literature, software must be made available to editors and reviewers. We strongly encourage code deposition in a community repository (e.g. GitHub). See the Nature Research guidelines for submitting code & software for further information.

Data

Policy information about availability of data

All manuscripts must include a data availability statement. This statement should provide the following information, where applicable:

- Accession codes, unique identifiers, or web links for publicly available datasets
- A list of figures that have associated raw data
- A description of any restrictions on data availability

The data that support the results of this study are available from the corresponding author upon reasonable request. The data are not publicly available because they contain information that might compromise the privacy of the research participants.

Field-specific reporting

Please select the one below that is the best fit for your research. If you are not sure, read the appropriate sections before making your selection.

- [x] Life sciences
- [ ] Behavioural & social sciences
- [ ] Ecological, evolutionary & environmental sciences

For a reference copy of the document with all sections, see nature.com/documents/nr-reporting-summary-flat.pdf

Life sciences study design

All studies must disclose on these points even when the disclosure is negative.

Sample size	No sample size calculation was performed. Observational study based on existing data.
Data exclusions	Data from patients with schizoaffective disorder or bipolar disorder were excluded.
Replication	The raw data used in this study were collected from existing electronic databases.
Randomization	Not relevant. Observational study based on retrospective data.
Blinding	Not relevant. Observational study based on retrospective data.

Reporting for specific materials, systems and methods

We require information from authors about some types of materials, experimental systems and methods used in many studies. Here, indicate whether each material, system or method listed is relevant to your study. If you are not sure if a list item applies to your research, read the appropriate section before selecting a response.

Materials & experimental systems

n/a	Involved in the study	
x		Antibodies
x		Eukaryotic cell lines
x		Palaeontology and archaeology
x		Animals and other organisms
	x	Human research participants
x		Clinical data
x		Dual use research of concern

Methods

n/a	Involved in the study	
x		ChIP-seq
x		Flow cytometry
x		MRI-based neuroimaging

Human research participants

Policy information about studies involving human research participants

Population characteristics	This study included 1458 patients (1431 patients with schizophrenia and 27 with schizophreniform disorder) who were prescribed oral antipsychotic medications. The mean ages (years) in each antipsychotic group were as follows: 32.2 for amisulpride; 28.8 for aripiprazole; 30.8 for clozapine; 33.3 for haloperidol; 32.3 for olanzapine; 31.3 for paliperidone; 33.8 for quetiapine; 32.3 for risperidone; and 28.7 for ziprasidone.
Recruitment	The study population included all patients who were treated in the inpatient and/or outpatient setting at SNUH from March 1, 2005, to February 28, 2014, with a diagnosis of schizophrenia or schizophreniform disorder. Potential biases include lack of clinical information and structured assessments. The real-world setting also implies a risk of sample selection bias and unmeasured confounding factors.
Ethics oversight	Approval from the Institutional Review Board at SNUH was obtained prior to collecting and analyzing the data. Written informed consent is not required for CDW-based studies using anonymized data.

Note that full information on the approval of the study protocol must also be provided in the manuscript.
